# A Focal Length Calibration Method for Vision Measurement Systems Based on Multi-Feature Composite Variable Weighting

**DOI:** 10.3390/s25226873

**Published:** 2025-11-11

**Authors:** Enshun Lu, Xiaofeng Li, Fangjing Yang, Daode Zhang, Xing Sun

**Affiliations:** 1 Institute of Agricultural Machinery Engineering Design and Research, Hubei University of Technology, Wuhan 430068, China; gundamresearcher@163.com (E.L.); jellyli0506@163.com (X.L.); hgzdd@126.com (D.Z.); 2 The Library of Wuhan University of Technology, Wuhan 430070, China; 3 National Key Laboratory of Agricultural Equipment Technology, Chinese Academy of Agricultural Mechanization Sciences Group Co., Ltd., Beijing 100083, China; sun_xing2010@163.com

**Keywords:** close-range photogrammetry, camera calibration, error correction, geometric constraints, composite weighting

## Abstract

Existing focal length calibration methods rely on predefined calibration fields or control point networks, which are unsuitable for real-time applications with variable zoom in industrial and agricultural environments. This paper proposes a method based on global scanning principles and geometric constraints, eliminating control points and using symmetric features. A spatial weighting strategy optimizes redundant measurements by integrating optical distortion and the spatial distribution of measured points, enhancing accuracy. Experimental results show that the method achieves micron-level calibration precision, significantly improving visual measurement system accuracy under complex zoom conditions.

## 1. Introduction

In frontline engineering applications, target objects are often characterized by large variations in distance and significant spatial scale differences. Such scenarios typically demand imaging systems with a large depth of field, necessitating frequent focusing and zooming operations [1]. These operations inevitably cause substantial changes in the camera’s focal length, rendering pre-calibrated parameters ineffective. Moreover, vision systems are commonly mounted on high-dynamic platforms such as industrial robots, agricultural machinery, or mobile inspection devices. During operation, these platforms are prone to intense vibrations, which may lead to subtle yet critical shifts in the relative positions of camera lens elements—directly affecting the stability of the focal length [2]. Over time or with prolonged equipment movement, vibration-induced focal length drift can accumulate, amplifying errors and severely compromising measurement accuracy [3,4]. Therefore, there is an urgent need for systems capable of dynamically responding to on-site disturbances and updating calibration parameters in real time [5,6].

Again, conventional focal length calibration methods generally depend on known three-dimensional control points or dedicated calibration setups [7,8,9]. These methods are not only complex to deploy and constrained by specific environments, but also poorly suited for scenarios where the target is inaccessible or where control points cannot be physically arranged [10,11]. In applications such as outdoor agricultural surveying or high-temperature and high-risk industrial inspections, this reliance on control points becomes a critical bottleneck that limits the applicability of such methods.

To address the aforementioned issues, this paper proposes a control-point-free focal length calibration method based on geometric symmetry constraints and the principle of global scanning (hereafter referred to as the traditional algorithm) [12].

## 2. Global Scanning Principle

**Figure 1:**
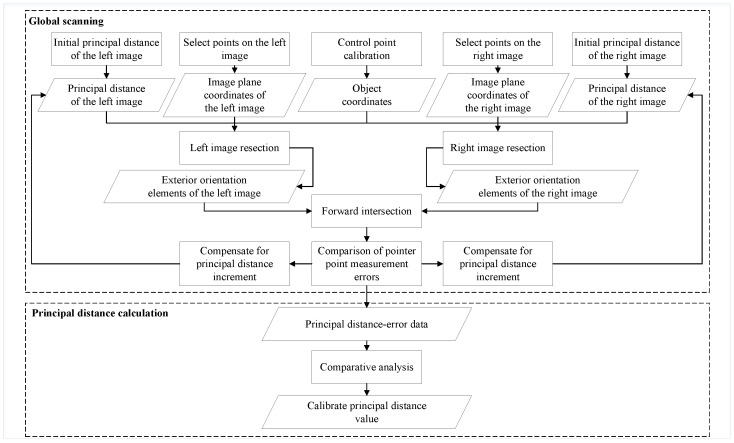
Schematic diagram of the global scanning principle.

Based on the collinearity Equation (1), a global scan is performed for focal length calibration.The schematic diagram of the global scanning principle is shown in Figure 1.

(1) $$\left[\begin{array}{c} v_{x}\\ v_{y} \end{array}\right]=A\left[\begin{array}{c} \Delta X_{s}\\ \Delta Y_{s}\\ \Delta Z_{s}\\ \Delta \omega \\ \Delta \varphi \\ \Delta \kappa \end{array}\right]+B\left[\begin{array}{c} \Delta X\\ \Delta Y\\ \Delta Z \end{array}\right]-\left[\begin{array}{c} l_{x}\\ l_{y} \end{array}\right].$$

where: $A=\left[\begin{array}{cccccc} O_{11} & O_{12} & O_{13} & O_{14} & O_{15} & O_{16}\\ O_{21} & O_{22} & O_{23} & O_{24} & O_{25} & O_{26} \end{array}\right]$; $B=\left[\begin{array}{ccc} -O_{11} & -O_{12} & -O_{13}\\ -O_{21} & -O_{22} & -O_{23} \end{array}\right]$; where:

$\;O_{11}=\frac{\left[a_{1}\cdot f+a_{3}\cdot \left(x-x_{0}\right)\right]}{\bar{Z}}$; $O_{12}=\frac{\left[b_{1}\cdot f+b_{3}\cdot \left(x-x_{0}\right)\right]}{\bar{Z}}$;

$\;O_{13}=\frac{\left[c_{1}\cdot f+c_{3}\cdot \left(x-x_{0}\right)\right]}{\bar{Z}}$;

$\;O_{14}=a_{2}\cdot \frac{{\left(x-x_{0}\right)}^{2}}{f}-a_{1}\cdot \frac{\left(x-x_{0}\right)\left(y-y_{0}\right)}{f}+a_{3}\cdot \left(y-y_{0}\right)+a_{2}\cdot f$;

$\;O_{15}=cos\kappa \cdot \frac{{\left(x-x_{0}\right)}^{2}}{f}-sin\kappa \cdot \frac{\left(x-x_{0}\right)\left(y-y_{0}\right)}{f}+f\cdot cos\kappa$; $O_{16}=y-y_{0}$;

$\;O_{21}=\frac{\left[a_{2}\cdot f+a_{3}\cdot \left(x-x_{0}\right)\right]}{\bar{Z}}$; $O_{22}=\frac{\left[b_{2}\cdot f+b_{3}\cdot \left(x-x_{0}\right)\right]}{\bar{Z}}$;

$\;O_{23}=\frac{\left[c_{2}\cdot f+c_{3}\cdot \left(x-x_{0}\right)\right]}{\bar{Z}}$;

$\;O_{24}=-a_{1}\cdot \frac{{\left(y-y_{0}\right)}^{2}}{f}+a_{2}\cdot \frac{\left(x-x_{0}\right)\cdot \left(y-y_{0}\right)}{f}-a_{3}\cdot \left(x-x_{0}\right)-a_{1}\cdot f$;

$\;O_{25}=-sin\kappa \cdot \frac{{\left(y-y_{0}\right)}^{2}}{f}+cos\kappa \cdot \frac{\left(x-x_{0}\right)\left(y-y_{0}\right)}{f}+f\cdot sin\kappa$; $O_{26}=-\left(x-x_{0}\right)$;

$\;a_{1}=cos\varphi cos\kappa -sin\varphi sin\omega sin\kappa$; $a_{2}=-cos\varphi sin\kappa -sin\varphi sin\omega cos\kappa$; $a_{3}=-sin\varphi cos\omega$;

$\;b_{1}=cos\omega sin\kappa$; $b_{2}=cos\omega cos\kappa$; $b_{3}=-sin\omega$;

$\;c_{1}=sin\varphi cos\kappa +cos\varphi sin\omega sin\kappa$; $c_{2}=-sin\varphi sin\kappa +cos\varphi sin\omega cos\kappa$; $c_{3}=cos\varphi cos\omega$;

$\;l_{x}=x-\left(x\right)$; $l_{y}=y-\left(y\right)$.

Table 1 provides the physical interpretations of the symbols in Equation (1).

### 2.1. Single-Position Focal Length Scanning Calibration Model

Let the parameters for a given pointer position be defined as follows:

Nominal focal length: $f_{bc}$; Zoom range of the lens: $\left[-f_{m},f_{m}\right]$; Number of calibration iterations: *n*; Calibration step size: $step=\frac{2\cdot f_{m}}{n}$.

Then, the single-position focal length scanning calibration model can be expressed as:

(2) $$qf=\{\Delta_{f_{1}},\Delta_{f_{2}},\ldots ,\Delta_{f_{\left(n-1\right)}},\Delta_{f_{n}}\}.$$

where i=1,2,3,…,n−1,n; $\Delta_{f_{i}}$: error of the *i*-th iteration.

If the minimum value corresponding to qf is:

(3) $$qf_{min}=\Delta_{f_{i}},\;1\leq i\leq n.$$

Then, the calibrated focal length value $f_{i}$ corresponding to the single-position focal length scanning calibration model is:

(4) $$f_{i}=f_{bc}-f_{m}+\frac{2\cdot f_{m}}{n}\cdot i.$$

### 2.2. Multi-Position Focal Length Scanning Calibration Model

By increasing redundant data, the focal length calibration error caused by random errors in the measurement values used as references can be minimized as much as possible.

Let there be *k* pointer positions configured for the same camera–lens combination.

Then, based on the same number of iterations and step size, *k* single-position focal length scanning calibration models can be obtained through computation:

(5) $$\begin{array}{c} qf_{1}=\left\{\Delta_{f_{11}},\Delta_{f_{12}},\ldots ,\Delta_{f_{1(n-1)}},\Delta_{f_{1n}}\right\}\\ qf_{2}=\left\{\Delta_{f_{21}},\Delta_{f_{22}},\ldots ,\Delta_{f_{2(n-1)}},\Delta_{f_{2n}}\right\}\\ \ldots \\ qf_{k}=\left\{\Delta_{f_{k1}},\Delta_{f_{k2}},\ldots ,\Delta_{f_{k(n-1)}},\Delta_{f_{kn}}\right\} \end{array}.$$

In this case, to select the focal length with the minimum error, it is necessary to combine the errors $\Delta_{f_{ki}}$ from each iteration at all pointer positions into a comprehensive error set QF. This set is called the multi-position focal length scanning calibration model:

(6) $$QF=\left\{\sum_{h=1}^{k}\Delta_{f_{h1}},\sum_{h=1}^{k}\Delta_{f_{h2}},\ldots ,\sum_{h=1}^{k}\Delta_{f_{h(n-1)}},\sum_{h=1}^{k}\Delta_{f_{hn}}\right\}.$$

Let the minimum value corresponding to QF be:

(7) $$QF_{min}=\sum_{h=1}^{k}\Delta_{f_{hi}},\;1\leq i\leq n.$$

Then, the calibrated focal length value $f_{j}$ corresponding to the multi-position focal length scanning calibration model is:

(8) $$f_{j}=f_{bc}-f_{m}+\frac{2\cdot f_{m}}{n}\cdot i.$$

## 3. Methodology

### 3.1. Focal Length Verification Algorithm Based on Geometric Symmetry Features

In the aforementioned traditional verification algorithms, the focal length verification involves comparing the computed coordinates of the pointer points with their actual coordinates [12]. This requires obtaining the actual object space coordinates of the verification points in advance, typically measured using precision instruments such as total stations.However, this approach is costly and inefficient. Research indicates that almost all mechanical structures possess symmetry [13], which can be exploited to transform the traditional verification based on pointer point object space coordinates into a symmetry-based verification, thereby enabling correction of the focal length [14,15].

The illustration shows a schematic diagram of geometric symmetry constraints.

**Figure 2:**
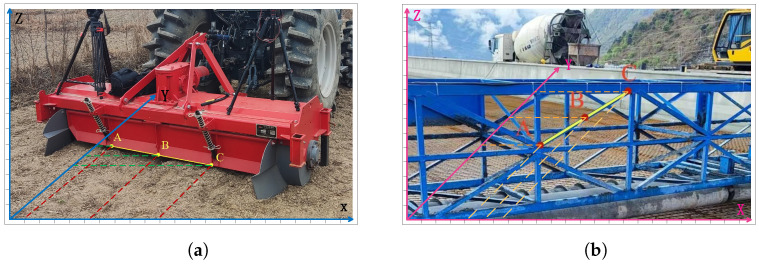
Symmetry cases: (**a**) Agricultural machinery symmetrical structure. (**b**) Symmetrical structure at bridge construction site.

The points A, B, and C in the Figure 2 are referred to as pointer points, and the symmetric constraint formed by these three points is referred to as a pointer point configuration. In the illustrated coordinate system, the coordinates of points $A(x_{a},y_{a},z_{a})$ and $C(x_{c},y_{c},z_{c})$ are symmetric with respect to point $B(x_{b},y_{b},z_{b})$.

(9) $$\left\{\begin{array}{c} \frac{x_{a}+x_{c}}{2}=x_{b}\\ \frac{y_{a}+y_{c}}{2}=y_{b}\\ \frac{z_{a}+z_{c}}{2}=z_{b} \end{array}\right.$$

According to Equation (1):

$$\Delta_{f_{i}}=\sqrt{{\left(\frac{X_{1i}+X_{3i}}{2}-X_{2i}\right)}^{2}+{\left(\frac{Y_{1i}+Y_{3i}}{2}-Y_{2i}\right)}^{2}+{\left(\frac{Z_{1i}+Z_{3i}}{2}-Z_{2i}\right)}^{2}};$$

$X_{1i}$, $X_{2i}$, $X_{3i}$, $Y_{1i}$, $Y_{2i}$, $Y_{3i}$, $Z_{1i}$, $Z_{2i}$, $Z_{3i}$ represent the object-space coordinates of the pointer points calculated from Equation (6) based on the current (to-be-evaluated) focal length, and are used as reference data for evaluation.

### 3.2. Weighted Algorithm Based on the Lens Distortion Model

#### 3.2.1. Lens Distortion Model

To further improve the accuracy of the calibration results, a weighted algorithm based on the lens optical distortion model is proposed to optimize the calibration errors caused by lens optical distortion and manual point selection deviations.

Due to inevitable manufacturing and assembly errors in the production process of the optical lenses of measurement cameras, optical distortion will theoretically always occur in images captured by measurement cameras.Such optical distortion significantly interferes with the accuracy of photogrammetric systems [16]. Therefore, to improve the accuracy of photogrammetric results, it is essential to correct the optical distortion of measurement cameras [17].

The distortion errors of optical lenses mainly include radial distortion, decentering distortion, and thin prism distortion. According to the study by RICOLFE-VIALA C et al. [18], the distortion model of an optical lens can be divided into distortion models along the *x*-axis and the *y*-axis directions:

(10) $$\begin{array}{cc} \; & \delta_{x}=\delta_{jx}+\delta_{lx}+\delta_{bx}=k_{1}x\left(x^{2}+y^{2}\right)+\left[q_{1}\left(3x^{2}+y^{2}\right)+2q_{2}xy\right]+s_{1}\left(x^{2}+y^{2}\right),\\ \; & \delta_{y}=\delta_{jy}+\delta_{ly}+\delta_{by}=k_{2}y\left(x^{2}+y^{2}\right)+\left[q_{2}\left(3x^{2}+y^{2}\right)+2q_{1}xy\right]+s_{2}\left(x^{2}+y^{2}\right). \end{array}$$

**Figure 3:**
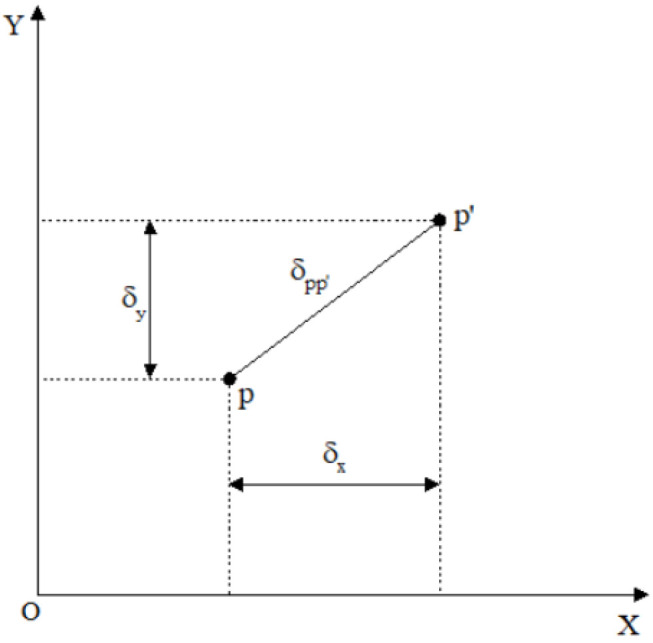
Spatial positional relationship within the image plane coordinate system.

Here, $\delta_{x}$ and $\delta_{y}$ represent the combined values of nonlinear distortion in the *x*-axis and *y*-axis directions of the image plane coordinate system, respectively; $\delta_{jx}$ and $\delta_{jy}$ denote the radial distortion parameters along the *x*-axis and *y*-axis directions, respectively; $\delta_{lx}$ and $\delta_{ly}$ denote the decentering distortion parameters along the *x*-axis and *y*-axis directions, respectively; $\delta_{bx}$ and $\delta_{by}$ denote the thin prism distortion parameters along the *x*-axis and *y*-axis directions, respectively; $k_{1}$ and $k_{2}$ are the radial distortion coefficients; $q_{1}$ and $q_{2}$ are the decentering distortion coefficients; and $s_{1}$ and $s_{2}$ are the thin prism distortion coefficients. The spatial positional relationship of $\delta_{x}$ and $\delta_{y}$ within the image plane coordinate system is shown in Figure 3.

In the figure, point *p* represents the theoretical position of the pixel, point $p^{\prime }$ represents the actual position of the pixel, and $\delta_{pp^{\prime }}$ denotes the distance between points *p* and $p^{\prime }$.

In practical engineering applications, although professional industrial cameras are rarely used, mid-to-high-end DSLRs such as the Canon 5Ds and their compatible original lenses are generally employed.Such equipment typically possesses relatively high manufacturing and assembly precision. Therefore, based on the theory proposed by RICOLFE-VIALA C et al., the optical distortion calculation model for this type of equipment can be moderately simplified [18], retaining the first-order radial distortion and second-order decentering distortion of the lens:

(11) $$\delta_{pp^{\prime }}=\sqrt{{\left(k_{1}u_{d}r_{d}^{2}+2q_{1}u_{d}\nu_{d}+q_{2}\left(r_{d}^{2}+2u_{d}^{2}\right)\right)}^{2}+{\left(k_{1}\nu_{d}r_{d}^{2}+2q_{2}u_{d}\nu_{d}+q_{1}\left(r_{d}^{2}+2\nu_{d}^{2}\right)\right)}^{2}}\cdot$$

where: $u_{d}=x-u_{0}$; $\nu_{d}=y-\nu_{0}$; $r_{d}=\sqrt{u_{d}^{2}+\nu_{d}^{2}}$; $\left(u_{0},\nu_{0}\right)$ denotes the coordinates of the camera principal point; $k_{1}$ and $k_{2}$ are the radial distortion parameters; $q_{1}$ and $q_{2}$ are the decentering distortion parameters.

#### 3.2.2. Weighting Method Based on Optical Distortion Parameters

Based on the above model, the coordinates of the pointer points for each position differ in the image, thus each has a distinct distortion value $\delta_{{\mathrm{pp}}^{\prime }}$. Furthermore, this distortion value is inversely proportional to the confidence level $CI_{1}$ of the image point:

(12) $$CI_{1}\propto \delta_{pp^{\prime }}^{-1}$$

For each point, there are 3 pointer points, and the corresponding distortion values $\delta_{\mathrm{pp}1^{\prime }}$, $\delta_{\mathrm{pp}2^{\prime }}$, and $\delta_{\mathrm{pp}3^{\prime }}$ are calculated respectively.The distortion value of a point in a single image is then given by:

(13) $$\delta_{pp^{\prime }}=\delta_{pp1^{\prime }}+\delta_{pp2^{\prime }}+\delta_{pp3^{\prime }}.$$

Suppose there are *k* inspection points and a total of *s* images are used in the calculation process.According to Equation (5), the optical distortion sets of all points in the multi-point focal length scanning calibration model can be obtained within each image plane coordinate system:

(14) $$\begin{array}{c} \delta_{1}=\left\{\delta_{pp^{\prime }11},\delta_{pp^{\prime }12},\cdots ,\delta_{pp^{\prime }1\left(s-1\right)},\delta_{pp^{\prime }1s}\right\}\\ \delta_{2}=\left\{\delta_{pp^{\prime }21},\delta_{pp^{\prime }22},\cdots ,\delta_{pp^{\prime }2\left(s-1\right)},\delta_{pp^{\prime }2s}\right\}\\ \cdots \\ \delta_{k}=\left\{\delta_{pp^{\prime }k1},\delta_{pp^{\prime }k2},\cdots ,\delta_{pp^{\prime }k\left(s-1\right)},\delta_{pp^{\prime }ks}\right\} \end{array}\cdot$$

According to Equation (14), the weight coefficients for each point can be calculated as follows:

(15) $$q_{1}={\left(\sum_{t=1}^{s}\delta_{pp^{\prime }1t}\right)}^{-1},q_{2}={\left(\sum_{t=1}^{s}\delta_{pp^{\prime }2t}\right)}^{-1},\cdots ,q_{k}={\left(\sum_{t=1}^{s}\delta_{pp^{\prime }kt}\right)}^{-1}\cdot$$

To improve the numerical stability of the model, the weight coefficients calculated by Equation (15) are normalized as follows:

(16) $$Q_{1}=\frac{{\left(\sum_{t=1}^{s}\delta_{pp^{\prime }1t}\right)}^{-1}}{\sum_{u=1}^{k}{\left(\sum_{t=1}^{s}\delta_{pp^{\prime }ut}\right)}^{-1}},Q_{2}=\frac{{\left(\sum_{t=1}^{s}\delta_{pp^{\prime }2t}\right)}^{-1}}{\sum_{u=1}^{k}{\left(\sum_{t=1}^{s}\delta_{pp^{\prime }ut}\right)}^{-1}},\cdots ,Q_{k}=\frac{{\left(\sum_{t=1}^{s}\delta_{pp^{\prime }kt}\right)}^{-1}}{\sum_{u=1}^{k}{\left(\sum_{t=1}^{s}\delta_{pp^{\prime }ut}\right)}^{-1}}\cdot$$

By substituting the weight coefficients $Q_{1}$, $Q_{2}$, ⋯, $Q_{k}$ into Equation (6) of the multi-point focal length scanning calibration model, the weighted multi-point focal length scanning calibration model is obtained:

(17) $$QFQ=\left\{\sum_{h=1}^{k}Q_{h}\cdot \Delta_{f_{h1}},\sum_{h=1}^{k}Q_{h}\cdot \Delta_{f_{h2}},\cdots ,\sum_{h=1}^{k}Q_{h}\cdot \Delta_{f_{hn}}\right\}$$

Let the minimum value corresponding to QFQ be:

(18) $$QFQ_{min}=\sum_{h=1}^{k}Q_{h}\cdot \Delta_{f_{hqj}},\;1\leq qj\leq n.$$

Then, the calibrated focal length value corresponding to the weighted multi-point focal length scanning calibration model is given by $f_{qj}$:

(19) $$f_{qj}=f_{bc}-f_{m}+\frac{2\cdot f_{m}}{n}\cdot qj$$

### 3.3. The Weighting Algorithm Based on the Spatial Distance to the Target Point

After obtaining the internal and external parameters of the camera, the object-space coordinates of the target point can be calculated using forward intersection.To further refine the focal length, once the object-space coordinates of the target point are derived using the focal length estimated by the traditional algorithm, different weights $Q^{\prime }$ are assigned to the pointer points based on the distance from the target point to the pointer point ($D_{dd}$) and the distance from the target point to the line formed by the pointer positions ($D_{dl}$). These weights are then averaged with the corresponding optical distortion weights *Q* to form a composite weight $Q_{f}$.

#### 3.3.1. Weighting Based on the Distance Between the Target Point and the Pointer Points

**Figure 4:**
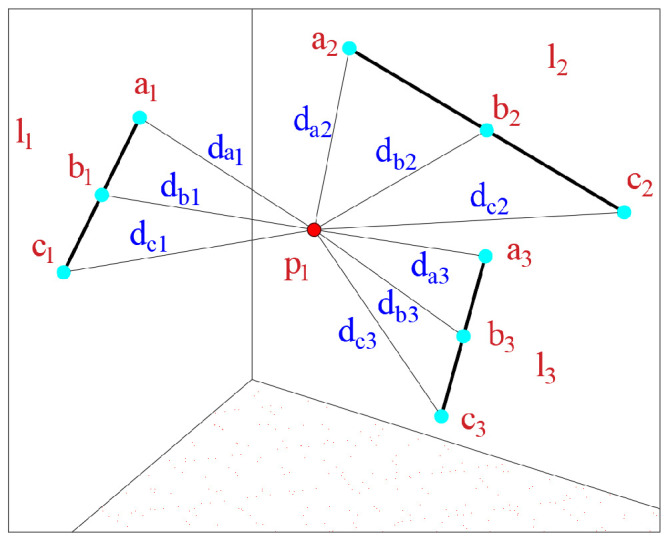
Schematic diagram of the distance between the target point and the pointer points.

As shown in Figure 4, let $p_{1}$ be the target point to be measured.Based on the aforementioned algorithm, its coordinates are computed as $p_{1}\left(x_{1},y_{1},z_{1}\right)$. Let $l_{h}$ be the line passing through the pointer points $a_{h},b_{h},c_{h}$, whose coordinates are calculated as $a_{h}\left(x_{ah},y_{ah},z_{ah}\right)$, $b_{h}\left(x_{bh},y_{bh},z_{bh}\right)$, and $c_{h}\left(x_{ch},y_{ch},z_{ch}\right)$, respectively. The distances from $p_{1}$ to $a_{h},b_{h},c_{h}$ are denoted by $d_{ah},d_{bh},d_{ch}$, where h=1,2,…,k. Then:

(20) $$D_{ddh}=d_{ah}+d_{bh}+d_{ch}$$

The confidence level $CI_{2}$ of $l_{u}$ is inversely proportional to the distance:

(21) $$CI_{2}\propto D_{ddh}^{-1}$$

Then, the weight of the pointer point $Q_{ddh}$ satisfies:

(22) $$Q_{dd1}=D_{dd1}^{-1},Q_{dd2}=D_{dd2}^{-1},\cdots ,Q_{ddk-1}=D_{ddk-1}^{-1},Q_{ddk}=D_{ddk}^{-1}$$

To improve the numerical stability of the model, the weights are normalized as follows:

(23) $$Q_{dd1}^{\prime }=\frac{D_{dd1}^{-1}}{\sum_{h=1}^{k}D_{ddh}^{-1}},Q_{dd2}^{\prime }=\frac{D_{dd2}^{-1}}{\sum_{h=1}^{k}D_{ddh}^{-1}},\cdots ,Q_{ddk-1}^{\prime }=\frac{D_{ddk-1}^{-1}}{\sum_{h=1}^{k}D_{ddh}^{-1}},Q_{ddk}^{\prime }=\frac{D_{ddk}^{-1}}{\sum_{h=1}^{k}D_{ddh}^{-1}}$$

#### 3.3.2. Weighting Based on the Distance from the Target Point to the Pointer Points’ Line

**Figure 5:**
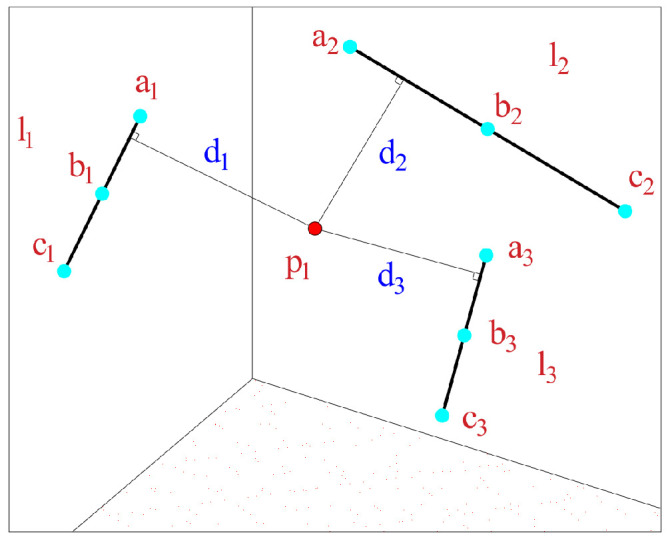
Schematic diagram of the distance from the target point to the pointer points’ line.

As shown in Figure 5, given the coordinates of $a_{u}$ and $c_{u}$ as $\left(x_{au},y_{au},z_{au}\right)$ and $\left(x_{cu},y_{cu},z_{cu}\right)$, respectively, the line $l_{u}$ is fitted based on $a_{u}$ and $c_{u}$. The distance from the target point $p_{1}\left(x_{1},y_{1},z_{1}\right)$ to the pointer points’ line $l_{u}$ is denoted as $d_{u}$, where u=1,2,⋯,k.

Due to the presence of lens optical distortion, the confidence level $CI_{3}$ of each inspection point is inversely proportional to the distance $d_{u}$:

(24) $$CI_{3}\propto d_{u}^{-1}.$$

Then, the weight of the pointer point $Q_{dl}$ satisfies:

(25) $$Q_{dl1}=d_{1}^{-1},Q_{dl2}=d_{2}^{-1},\cdots ,Q_{dlk-1}=d_{k-1}^{-1},Q_{dlk}=d_{k}^{-1}$$

To improve the numerical stability of the model, the weight coefficients calculated by Equation (25) are normalized as follows:

(26) $$Q_{dl1}^{\prime }=\frac{d_{1}^{-1}}{\sum_{h=1}^{k}d_{h}^{-1}},Q_{dl2}^{\prime }=\frac{d_{2}^{-1}}{\sum_{h=1}^{k}d_{h}^{-1}},\cdots ,Q_{dlk-1}^{\prime }=\frac{d_{k-1}^{-1}}{\sum_{h=1}^{k}d_{h}^{-1}},Q_{dlk}^{\prime }=\frac{d_{k}^{-1}}{\sum_{h=1}^{k}d_{h}^{-1}}.$$

### 3.4. Based on Composite Weighting

By taking the average of the weights $Q_{ddh}^{\prime }$ and $Q_{dlh}^{\prime }$, the composite spatial distance weight $Q_{h}^{\prime }$ for the pointer point is obtained:

(27) $$Q_{h}^{\prime }=\frac{Q_{ddh}^{\prime }+Q_{dlh}}{2}$$

The normalized final optical distortion composite weight $Q_{hh}$ is obtained by normalizing the composite spatial distance weight $Q_{h}^{\prime }$ and the optical distortion weight $Q_{h}$ of each pointer point.Substituting $Q_{hh}$ into Equation (17) of the weighted multi-point focal length scanning calibration model yields the spatial composite weighted multi-point focal length scanning calibration model:

(28) $$QFQ^{\prime }=\left\{\sum_{h=1}^{k}Q_{hh}\cdot \Delta_{f_{h1}},\sum_{h=1}^{k}Q_{hh}\cdot \Delta_{f_{h2}},\cdots ,\sum_{h=1}^{k}Q_{hh}\cdot \Delta_{f_{hn}}\right\}$$

Let the minimum value corresponding to $QFQ^{\prime }$ be:

(29) $$QFQ_{min}^{\prime }=\sum_{h=1}^{k}Q_{hh}\cdot \Delta f_{hq^{\prime }j},\;1\leq q^{\prime }j\leq n.$$

Then, the calibrated focal length value corresponding to the spatial weighted multi-point focal length scanning calibration model is given by $f_{q^{\prime }j}$:

(30) $$f_{q^{\prime }j}=f_{bc}-f_{m}+\frac{2\cdot f_{m}}{n}\cdot q^{\prime }j$$

## 4. Experimental Validation

### 4.1. Experimental Procedure

**Figure 6:**
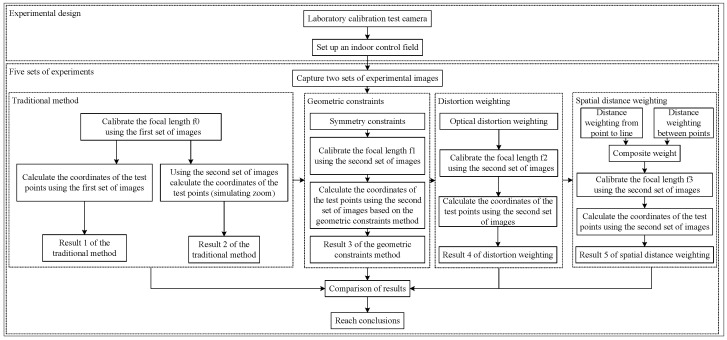
Schematic diagram of the experimental procedure.

To validate the effectiveness of incorporating geometric constraints as pointer points and compensating for the focal length, the traditional algorithm was set as the control group. To further verify the effectiveness of distortion weighting and spatial distance weighting, the focal length was corrected according to different weighting values. The evaluation was based on the 3D coordinates of the test points calculated using the corresponding focal lengths, with the 3D coordinates obtained from total station calibration serving as the reference standard to measure the errors. The experimental procedure is shown in Figure 6. A total of five groups of experiments were designed, where test images were captured at different locations. Each group included the same six test points, resulting in five sets of data from different algorithms. The comprehensive analysis of these five groups of experimental results led to the conclusions.

### 4.2. Matlab Programming

**Figure 7:**
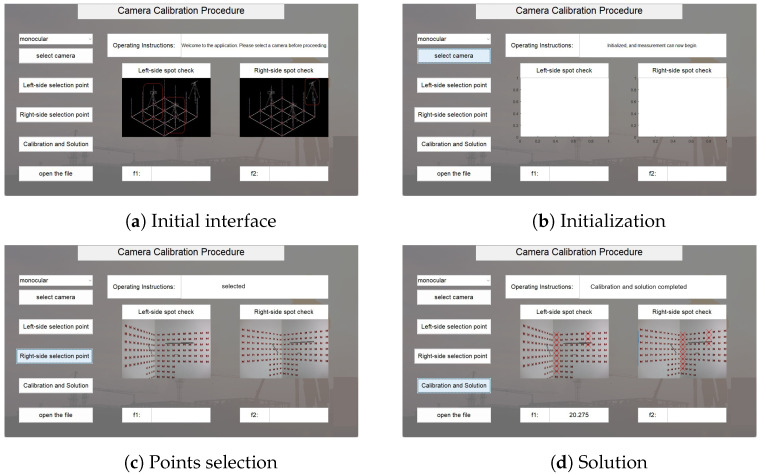
GUI of the MATLAB2015b program.

Based on the mathematical model described in the methodology and the experimental procedure shown in Figure 7, a Matlab program was developed for the experiment. The graphical user interface (GUI) and its operation process is shown in the figure.

The computer is equipped with a 13th Gen Intel(R) Core(TM) i5-13490F processor (2.5 GHz), 10 cores and 16 logical processors, and 32.0 GB of physical memory (RAM).

### 4.3. Experimental Design

#### 4.3.1. Calibrate the Test Camera

The camera used in this experiment is a high-resolution DSLR, the Canon EOS 5Ds, Canon Inc., Japan equipped with a Canon EF 50mm f/1.8 STM lens. The technical specifications of the camera are shown in Table 2.

The camera calibration tool used in this experiment is the MATLAB Camera Calibrator Toolbox 6.6. The calibration results of the camera are shown in Table 3.

In the table: *f* represents the calibrated focal length, which will be used as the initial calibration value for the traditional method; Δx and Δy are the offsets of the principal point in the image plane coordinate system; $k_{1}$ and $k_{2}$ are the radial distortion parameters of the camera; $q_{1}$ and $q_{2}$ are the tangential distortion parameters; and $s_{1}$ and $s_{2}$ represent the thin prism distortion parameters.

#### 4.3.2. Set Up the Indoor Control Field

**Figure 8:**
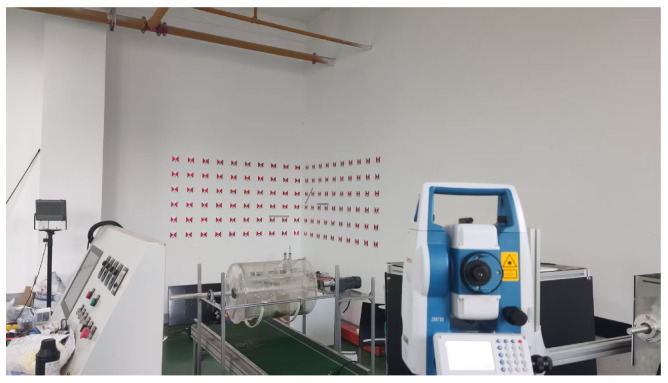
Indoor control field.

To facilitate the verification of the algorithms proposed in this paper under laboratory conditions, an indoor control field was designed, as shown in Figure 8. The following conditions are satisfied:

- A total of 120 three-dimensional control point targets were established;
- The control points are evenly distributed and extend along all three coordinate axes;
- Sufficient shooting space was reserved for the camera.

The 3D coordinates of the control points were obtained in advance using a reflectorless total station.

**Figure 9:**
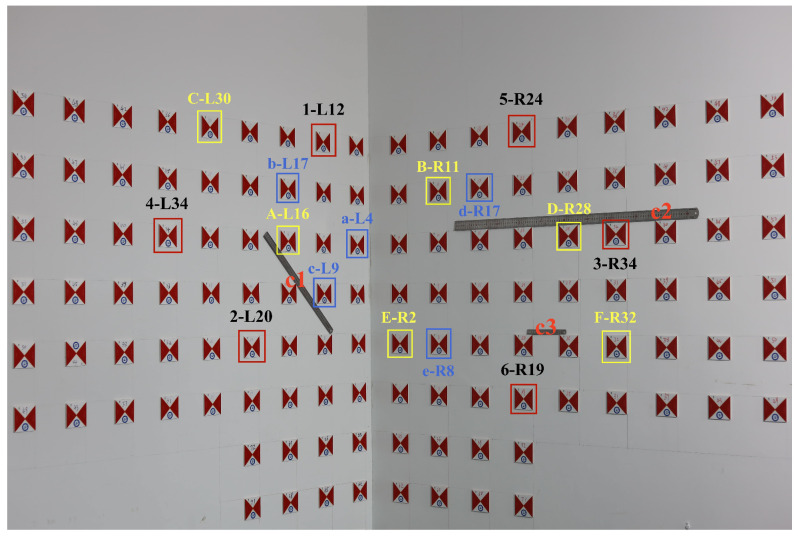
Guidelines for selecting test points.

As shown in Figure 9, in this experiment: Points 1-L12, 2-L20, 3-R34, 4-L34, 5-R24, and 6-R19 serve as control points; points a-L4, b-L17, c-L9, d-R17, and e-R8 are used as indicator points and are only involved in the computation of the traditional method; c1, c2, and c3 are steel rulers measuring 50 mm, 15 mm, and 1000 mm, respectively, serving as geometrically symmetric constraint points; A-L16, B-R11, C-L30, D-R28, E-R2, and F-R32 are designated as test points.

The precise object-space coordinates of all points were measured using a total station and are listed in Table 4.

#### 4.3.3. Capture of Experimental Images

**Figure 10:**
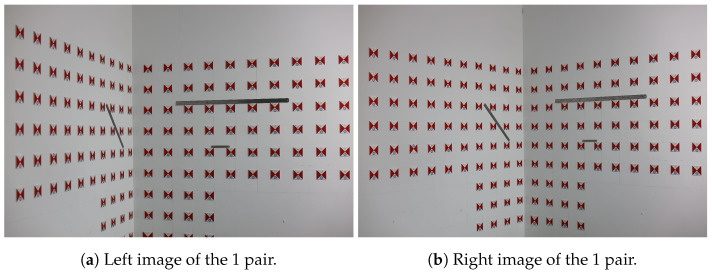
Image pairs from Experiment Group 1, pair 1.

**Figure 11:**
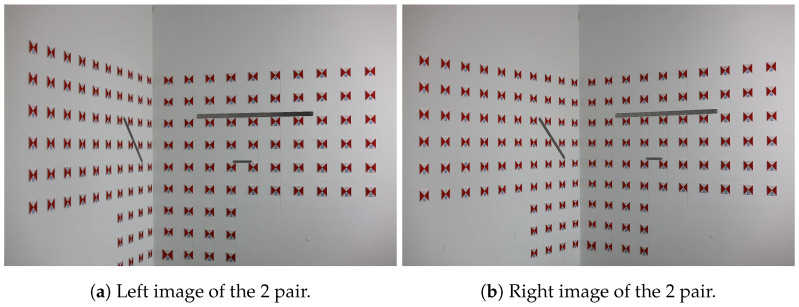
Image pairs from Experiment Group 1, pair 2.

According to classical photogrammetric methods, at least one pair of left and right images is required to calculate the interior and exterior orientation elements. As shown in the experimental procedure in Figure 6, the first image pair is taken after focusing, followed by a second image pair captured after repositioning and refocusing. Due to this process, the first and second image pairs have different focal lengths. The 3D coordinates of the test points in the second image pair are calculated using the focal length of the first image pair, simulating focal length variations caused by refocusing or vibration in real engineering scenarios. The images from the first sets of experiments are shown in Figure 10 and Figure 11.

### 4.4. Calibration and Solution

During the calibration process, the traditional method sets the focal length search range to 20–80 mm.

In the subsequent algorithm, the search range was adjusted to 6 mm with 12,000 iterations. Since Newton’s iteration is sensitive to the initial value, the calibrated focal lengths $f_{0}$, $f_{1}$, $f_{2}$, and $f_{3}$ were sequentially used as the initial values for focal length calibration in the following steps.

The lens used in this experiment is Canon EF 50mm f/1.8 STM. According to the manufacturer’s specifications and our previous experimental experience, the principal distance variation of this lens during the focusing process does not exceed ±3 mm. Therefore, the iterative parameters were set accordingly.

The number of iterations is a key factor affecting the accuracy of the algorithm described in this paper. We generally set it to 12,000 iterations for:

- Given the typical configuration of laptops used by on-site engineers, 12,000 iterations can be completed within an acceptable time—usually within 200 s;
- Once the single-step precision reaches 0.001 mm, further reducing this parameter contributes little to the overall solution accuracy.

The image plane coordinates of each point in first experimental image groups are listed in Table 5 and Table 6, where points a, b, c, d, e, f, g, h, and i represent the left endpoint, midpoint, and right endpoint of the symmetry-constrained points, respectively.

As shown in Table 7, $f_{0}$, $f_{1}$, $f_{2}$, $f_{3}$, and $f_{4}$ represent, respectively: the manually set initial focal length; the focal length calculated from the first set of images using the traditional algorithm; the focal length calculated from the second set of images using the geometric constraint algorithm; the focal length calculated from the second set of images using the weighted algorithm incorporating optical distortion; and the focal length calculated from the second set of images using the composite weighting algorithm incorporating spatial distance.

The Table 8, Table 9, Table 10 and Table 11 shows the exterior orientation elements of each image in the first set of experiments.

Based on the image plane coordinates and the interior and exterior orientation parameters obtained from calibration, the coordinates of the test points were computed. The results are shown in Table 12, Table 13, Table 14, Table 15 and Table 16.

## 5. Results and Discussion

**Figure 12:**
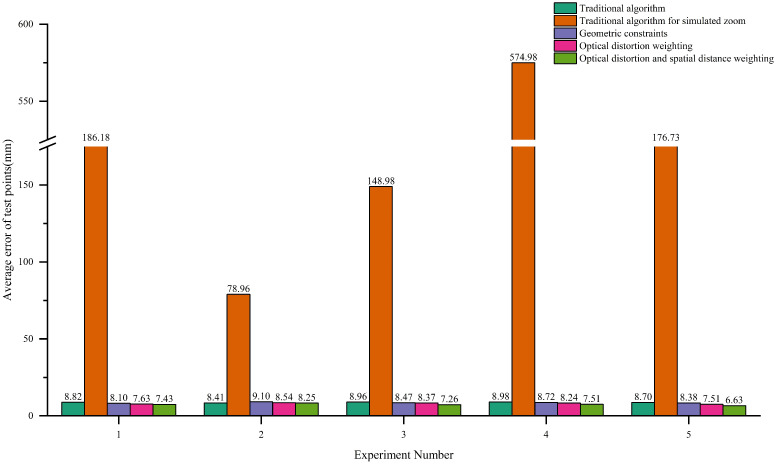
Bar chart of mean errors.

As shown in Figure 12, the average point error of each algorithm across the five test groups is presented. It can be seen that the traditional algorithm yields an average error of 8.78 mm. When using an incorrect focal length after the focal length was changed, the average error increases drastically to 233.17 mm, indicating a severe degradation in measurement accuracy. After recalculating the focal length using the algorithm proposed in this paper, the average error in each test group is significantly reduced. Furthermore, the measurement accuracy is further improved by introducing optical distortion weighting and spatial distance weighting.

**Figure 13:**
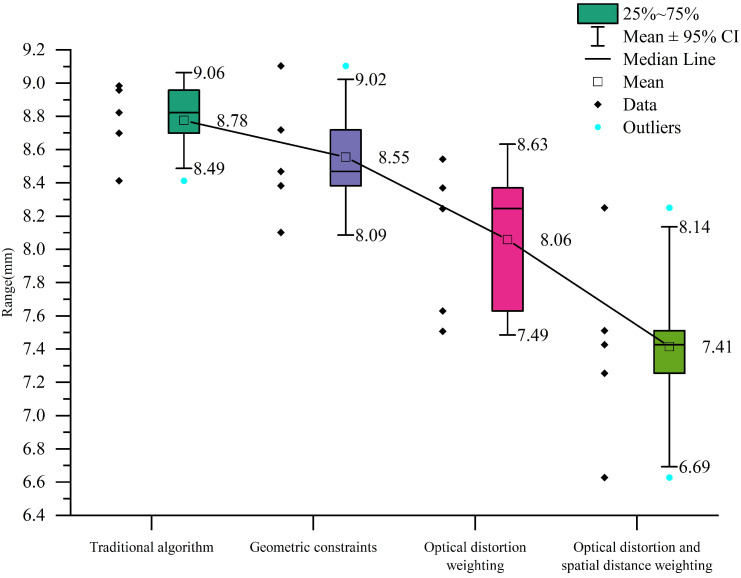
Box plot of mean errors.

As shown in Figure 13, the error comparison for each algorithm is presented. With iterative algorithmic optimization, the error gradually decreases from 8.78 mm to 7.41 mm, resulting in an overall accuracy improvement of approximately 15.6%. The relative improvement rates at each stage are 2.62%, 5.73%, and 8.07%, respectively.

In summary, the control-point-free focal length calibration method proposed in this paper not only overcomes the traditional algorithms’ reliance on control points and calibration fields, but also adapts well to complex, dynamic, and uncontrollable conditions commonly encountered in engineering applications. While maintaining system flexibility, it achieves micron-level calibration accuracy of the focal length, providing a reliable technical foundation for high-precision vision measurement in fields such as industry and agriculture.

## Figures and Tables

**Table 1:** Table of symbols.

Name	Explanation	Unit
$v_{x},v_{y}$	image point coordinate corrections	mm
(ω,φ,κ)	exterior orientation elements (orientation angles)	°
(x,y)	image-plane coordinates of the image point	mm
$\left(x_{0},y_{0}\right)$	image-plane coordinates of the principal point	mm
$\left(X_{s},Y_{s},Z_{s}\right)$	object-space coordinates of the camera center	mm
$\left(\bar{X},\bar{Y},\bar{Z}\right)$	image-space coordinates of the object point	mm
(x),(y)	approximate value from the previous iteration	mm
*f*	focal length	mm
(X,Y,Z)	object-space coordinates of the object point	mm
Δ	correction operator	

**Table 2:** Technical specifications of the test camera.

Parameter	Value	Unit
CCD resolution	8688 × 5792	pixels
Pixel size	4.14	μm

**Table 3:** Calibration results of the test camera.

Parameter	Value	Parameter	Value
*f*(mm)	54.39	$q_{1}$	−0.0007295
Δx(mm)	17.97	$q_{2}$	−0.0007047
Δy(mm)	11.30	$s_{1}$	0
$k_{1}$	−0.1495	$s_{2}$	0
$k_{2}$	−0.1458		

**Table 4:** Object-space coordinates of control points, indicator points, and test points.

Number	x (mm)	y (mm)	z (mm)	Number	x (mm)	y (mm)	z (mm)
1-L12	−1157	8496	1690	d-R17	−1758	8319	1489
2-L20	−897	8166	890	e-R8	−1602	8449	887
3-R34	−2215	7935	1290	A-L16	−1287	8645	1287
4-L34	−635	7861	1291	B-R11	−1604	8448	1488
5-R24	−1909	8192	1689	C-L30	−763	8014	1690
6-R19	−1907	8191	688	D-R28	−2062	8063	1289
a-L4	−1287	8645	1287	E-R2	−1450	8577	886
b-L17	−1027	8339	1492	F-R32	−2213	7934	889
c-L9	−1602	8449	887				

**Table 5:** First set of images, first group of image plane coordinates for the first experiment.

No.	$x_{l}$ (Pixel)	$y_{l}$ (Pixel)	$x_{r}$ (Pixel)	$y_{r}$ (Pixel)
1	2915.304446	4268.459152	3685.612012	4167.712359
2	2465.555500	2057.042761	3021.638436	2260.264324
3	6177.994276	3223.974262	6409.409706	3311.038932
4	1895.839224	3310.269331	2239.116946	3295.281711
5	5092.168316	4271.782926	5531.238414	4264.914888
6	5111.900364	1575.940862	5549.529875	1778.117378
a	3126.264742	3158.992650	4003.131816	3211.693944
b	2694.859231	3784.187451	3358.225485	3732.738076
c	2923.355872	2642.612996	3695.018932	2757.767444
d	4583.215765	3717.948734	5139.344651	3740.670620
e	4077.974894	2117.578605	4761.096607	2288.814240

**Table 6:** First set of images, second group of image plane coordinates for the first experiment.

No.	$x_{l}$ (Pixel)	$y_{l}$ (Pixel)	$x_{r}$ (Pixel)	$y_{r}$ (Pixel)
1	3014.894649	4174.054300	3534.511674	4105.041802
2	2568.849625	2151.149045	2920.416528	2246.963792
3	6006.319048	3206.436374	6220.335682	3250.511088
4	2028.906022	3285.729541	2204.155340	3261.390220
5	5019.210222	4177.894540	5355.045035	4175.430140
6	5024.626975	1690.513664	5363.001270	1778.841921
a	2663.243038	3271.599343	3052.664024	3263.793174
b	2854.251687	2811.443880	3316.293994	2845.845069
c	3035.032060	2371.887933	3565.704843	2448.340542
d	4295.433220	3336.269728	4748.092594	3354.009166
e	5462.684269	3364.295773	5743.590179	3396.980181
f	6698.026736	3392.742845	6847.065216	3445.642024
g	5089.444733	2319.666372	5420.034036	2384.448572
h	5260.355646	2321.877909	5566.425056	2384.534010
i	5431.061551	2323.431701	5715.034374	2385.162602

**Table 7:** Focal length.

No.	$f_{0}$	$f_{1}$	$f_{2}$	$f_{3}$	$f_{4_{1}}$	$f_{4_{2}}$	$f_{4_{3}}$	$f_{4_{4}}$	$f_{4_{5}}$	$f_{4_{6}}$
1		61.45	58.46	55.47	53.65	53.39	53.54	53.39	53.25	53.05
2		61.23	52.45	53.09	53.42	53.09	53.33	53.09	53.26	53.09
3	50	52.98	52.74	52.67	53.42	52.49	53.31	52.49	52.84	52.68
4		59.76	51.09	50.96	51.16	51.08	51.09	51.00	50.58	50.40
5		60.52	53.70	53.70	53.27	53.83	53.70	53.86	53.49	53.50

**Table 8:** External orientation element for the 1st group using the traditional algorithm.

No.	X (mm)	Y (mm)	Z (mm)	ω (°)	ϕ (°)	κ (°)
Left	408.18	3217.88	1020.24	−1.19	−4.79	−6.36
Right	−1385.05	2261.01	993.19	−1.55	0.28	−1.30

**Table 9:** External orientation elements for the 1st group using the geometric constraint algorithm.

No.	X (mm)	Y (mm)	Z (mm)	ω (°)	ϕ (°)	κ (°)
Left	325.63	2979.92	1031.79	−1.21	1.50	−62.90
Right	−1058.38	2369.11	1013.67	−1.65	877.75	945.29

**Table 10:** External orientation elements for the 1st group using the optical distortion algorithm.

No.	X (mm)	Y (mm)	Z (mm)	ω (°)	ϕ (°)	κ (°)
Left	267.29	3266.47	1046.40	−1.21	1.51	−69.18
Right	−1054.23	2667.91	1036.63	−1.49	1.30	−63.11

**Table 11:** External orientation elements of left and right images for test points A to F in the 1st group using composite weighting.

Test Point	No.	X (mm)	Y (mm)	Z (mm)	ω (°)	ϕ (°)	κ (°)
A	Left	233.06	3438.14	1055.77	−1.94	23.50	−34.62
	Right	−1051.07	2846.57	1049.27	−1.49	1.33	−69.36
B	Left	228.12	3462.93	1057.11	−1.20	1.51	−44.04
	Right	−1050.58	2872.35	1051.08	−1.65	61.03	−3.38
C	Left	231.08	3448.06	1056.31	−1.20	1.51	−69.18
	Right	−1050.87	2856.88	1050.00	−1.49	1.33	−69.36
D	Left	228.10	3463.03	1057.12	−1.94	4.65	−34.62
	Right	−1050.57	2872.45	1051.08	−1.65	73.59	9.19
E	Left	225.48	3476.20	1057.83	−1.20	7.79	−56.61
	Right	−1050.31	2886.16	1052.04	−1.65	−14.37	−85.06
F	Left	221.73	3495.05	1058.85	−1.20	3777.71	3725.87
	Right	−1049.93	2905.76	1053.41	4.80	−42.64	−245.28

**Table 12:** Calculation errors of test points using traditional algorithm in the first experiment.

No.	X	Y	Z	Distance Error	X Error	Y Error	Z Error
A-L16	−1026.45	8327.16	1291.06	6.30	3.45	5.16	1.06
B-R11	−1604.33	8460.12	1488.49	12.13	0.33	12.12	0.49
C-L30	−764.41	8012.07	1696.20	6.64	1.41	1.93	6.20
D-R28	−2064.24	8067.13	1289.43	4.72	2.24	4.13	0.43
E-R2	−1451.61	8594.38	891.37	18.26	1.61	17.38	5.37
F-R32	−2216.75	7937.13	889.17	4.89	3.75	3.13	0.17
Average error (mm)				8.82	2.13	7.31	2.29

**Table 13:** Calculation errors of test points using traditional algorithm in the first experiment (simulated zoom).

No.	X	Y	Z	Distance Error	X Error	Y Error	Z Error
A-L16	−991.56	8077.33	1270.39	247.46	31.44	244.67	19.61
B-R11	−1522.72	8233.91	1449.41	232.23	81.28	214.09	38.59
C-L30	−760.57	7832.44	1638.94	188.62	2.43	181.56	51.06
D-R28	−1986.06	8013.03	1273.37	92.24	75.94	49.97	15.63
E-R2	−1371.11	8303.76	910.50	285.46	78.89	273.24	24.50
F-R32	−2142.74	7934.28	899.75	71.08	70.26	0.28	10.75
Average error (mm)				186.18	56.71	160.64	26.69

**Table 14:** Calculation errors of test points using geometry-constrained algorithm in the first experiment.

No.	X	Y	Z	Distance Error	X Error	Y Error	Z Error
A-L16	−1025.03	8327.97	1290.68	6.35	2.03	5.97	0.68
B-R11	−1604.55	8460.66	1488.69	12.69	0.55	12.66	0.69
C-L30	−763.66	8013.00	1693.72	3.91	0.66	1.00	3.72
D-R28	−2063.52	8066.67	1289.00	3.97	1.52	3.67	0.00
E-R2	−1451.55	8595.64	889.82	19.09	1.55	18.64	3.82
F-R32	−2214.84	7935.82	888.86	2.59	1.84	1.82	0.14
Average error (mm)				8.10	1.36	7.29	1.51

**Table 15:** Calculation errors of test points using optical-distortion-incorporated geometry-constrained algorithm in the first experiment.

No.	X	Y	Z	Distance Error	X Error	Y Error	Z Error
A-L16	−1024.44	8327.76	1290.30	5.95	1.44	5.76	0.30
B-R11	−1606.11	8462.07	1489.08	14.27	2.11	14.07	1.08
C-L30	−763.65	8014.35	1692.00	2.13	0.65	0.35	2.00
D-R28	−2063.33	8065.94	1289.00	3.23	1.33	2.94	0.00
E-R2	−1452.85	8595.86	887.93	19.17	2.85	18.86	1.93
F-R32	−2213.00	7932.98	889.18	1.04	0.00	1.02	0.18
Average error (mm)				7.63	1.40	7.17	0.92

**Table 16:** Calculation errors of test points using composite weighted algorithm incorporating geometric constraints, optical distortion and spatial distance metrics in the first experiment.

No.	X	Y	Z	Distance Error	X Error	Y Error	Z Error
A-L16	−1024.04	8327.46	1289.88	5.56	1.04	5.46	0.12
B-R11	−1607.24	8463.10	1489.25	15.49	3.24	15.10	1.25
C-L30	−763.58	8014.70	1690.49	1.03	0.58	0.70	0.49
D-R28	−2062.95	8065.08	1289.06	2.29	0.95	2.08	0.06
E-R2	−1453.32	8595.11	886.32	18.41	3.32	18.11	0.32
F-R32	−2212.54	7935.71	889.20	1.78	0.46	1.71	0.20
Average error (mm)				7.43	1.60	7.19	0.40

## Data Availability

Data underlying the results presented in this paper are not publicly available at this time but may be obtained from the authors upon reasonable request.
